# An Atypical Age-Specific Pattern of Hepatocellular Carcinoma in Peru: A Threat for Andean Populations

**DOI:** 10.1371/journal.pone.0067756

**Published:** 2013-06-28

**Authors:** Stéphane Bertani, Pascal Pineau, Sebastian Loli, Julien Moura, Mirko Zimic, Eric Deharo, Eloy Ruiz

**Affiliations:** 1 Université de Toulouse 3, Unité de Pharmacochimie et Pharmacologie pour le Développement, UMR152, Toulouse, France; 2 Institut de Recherche pour le Développement, Unité de Pharmacochimie et Pharmacologie pour le Développement, UMR152, Lima, Peru; 3 Institut Pasteur, Institut National de la Santé et de la Recherche Médicale, Nuclear Organization and Oncogenesis Unit, U993, Paris, France; 4 Universidad Peruana Cayetano Heredia, Facultad de Ciencias, Laboratorios de Investigación y Desarrollo, Unidad de Bioinformática y Biología Molecular, Lima, Peru; 5 Institut de Recherche pour le Développement, Programme Andin de Recherche et de Formation sur la Vulnérabilité et les Risques en Milieu Urbain, Lima, Peru; 6 Instituto Nacional de Enfermedades Neoplásicas, Departamento de Cirugía en Abdomen, Lima, Peru; Seoul National University, Republic of Korea

## Abstract

**Background:**

In South America, the highest incidence of primary liver cancer is observed in Peru. However, national estimations on hepatocellular carcinoma incidence and mortality are approximated using aggregated data from surrounding countries. Thus, there is a lack of tangible information from Peru that impairs an accurate description of the local incidence, presentation, and outcomes of hepatocellular carcinoma. The present study attempts to fill this gap and assesses the clinical epidemiology of hepatocellular carcinoma in this country.

**Methods:**

A retrospective cohort study was conducted by analysing the medical charts of 1,541 patients with hepatocellular carcinoma admitted between 1997 and 2010 at the Peruvian national institute for cancer. The medical records including liver function, serologic status, and tumor pathology and stage were monitored. Statistical analyses were performed in order to characterize tumor presentation according to demographic features, risk factors, and regional origin.

**Results:**

Surprisingly, the age distribution of the patient population displayed bimodality corresponding to two distinct age-based subpopulations. While an older group was in keeping with the age range observed for hepatocellular carcinoma around the world, a younger population displayed an abnormally juvenile mean age of 25.5 years old. In addition, each subpopulation displayed age-specific pathophysiological and clinical characteristics.

**Conclusions:**

The analysis suggests two different age-specific natural histories of hepatocellular carcinoma in the Peruvian patient population. This otherwise unusual tumor process that is ongoing in younger patients leads to the hypothesis that there may be a Peru-endemic risk factor driving hepatocarcinogenesis in the local population.

## Introduction

Hepatocellular carcinoma (HCC), the main form of primary liver cancer, is the sixth most common malignancy and the third leading cause of tumor-related death in the world [1], [2]. Global clinical epidemiology of HCC defines a dominant patient profile corresponding grossly to males over 40 years old [3], [4]. The incidence rate of HCC has doubled worldwide during the last two decades, with nearly 85% of the recorded cases occurring in developing countries [1], [2], [5]. The greatest burden of HCC is borne in sub-Saharan Africa and eastern Asia, where chronic infection with hepatitis B virus (HBV) is highly endemic [1]–[6].

Reference reviews on the worldwide burden of HCC have constantly neglected to include the epidemiology of the disease in South America creating a gap between the existing epidemiological situation on the field and the global epidemics of HCC described in literature [2]–[5]. While the incidence rate of primary liver cancer in South America is considered low, the epidemiology of HCC on this continent displays intriguing characteristics. For example, the sex ratio of HCC is more balanced in South America than in any other regions of the world [1]. Nonetheless, with the exception of Brazil, a lack of information on primary liver cancers impairs an accurate description of HCC outcomes at both national and continental levels [1]. Incidence and mortality data for HCC in South America are often gathered from sparse cancer registries, and estimations are thus approximated using aggregated data from surrounding countries [1].

Peru is considered to have the highest incidence of primary liver cancer in South America [1]. Very few studies have been conducted concerning the clinical epidemiology of HCC among the Peruvian population; therefore our knowledge of the disease in Peru is lacking. To address this issue, we performed a retrospective study on Peruvian patients diagnosed with HCC between 1997 and 2010. Specifically, this study was designed to assess the clinical epidemiology of Peruvian patient population with HCC in a large well-characterized cohort.

## Methods

### 2.1. Study Design

The current study was conducted retrospectively within a cohort assembled by analysing the medical records of 1,541 patients admitted for HCC at the Peruvian national hospital for cancer (INEN) between January 1997 and December 2010. Written consent was given by the patients for their information to be stored in the Department of Cancer Statistics and Epidemiology of INEN, and used for research. Human Subjects committees at both the INEN (Peru) and the Institut Pasteur (France) approved this study.

Under the Peruvian Ministry of Health, INEN is the health care institution in charge of the management of neoplastic diseases at the national level [7]. As a public hospital, INEN treats patients regardless of age, sex, ethnicity, place of residence, economic status, and health care coverage. The centralization of the Peruvian health care system means that INEN serves as a national hub for neoplasm management and handles a large ratio of cancer cases from across the nation, providing a valuable environment for assessing the clinical-epidemiological context of HCC in Peru [7].

The vital records (i.e. sex, age, birthplace, place of residence, social situation, and family health history), personal medical history, liver function, and physiological, biochemical, and immunological status of patients with HCC were monitored for the duration of their hospital stay. The overall patient population was 1,541, corresponding to the whole patient population with HCC admitted at INEN between 1997 and 2010. However, due to the context of limited resources in Peru, INEN could not routinely examine all clinical specimens for every biomedical analysis, notably for hepatitis infection and tumor grading [8]. The opportunity of each biomedical analysis was then at the own discretion of the practitioner taking into account the patient’s best interests. The available cohorts for the different pathophysiological parameters analyzed from the initial 1,541 patient’s number are presented (Table 1).

**Table 1:** Patient characteristics at baseline.

		Number of patients(≤44 and >44 groups)	Number of patients (≤44 group)	Number of patients (>44 group)	*P*-value
**Overall cohort**		1,541 (100%)	778 (50.5%)	763 (49.5%)	–
**Age**	Mean±SEM	44.8±21.9	25.5±9.5	64.5±10.4	–
	Median	44	25	65	
	Range	[0–89]	[0–44]	[45–89]	
**Sex**	Male	942 (61.1%)	520 (66.8%)	422 (55.3%)	
	Female	599 (38.9%)	258 (33.2%)	341 (44.7%)	
	Total	1,541 (100%)	778 (100%)	763 (100%)	<0.0001*
**Tumor grade** †	G1	47 (13.7%)	22 (14.3%)	25 (13.2%)	
	G2	226 (65.7%)	86 (55.8%)	140 (73.7%)	
	G3	71 (20.6%)	46 (29.9%)	25 (13.2%)	
	Total	344 (100%)	154 (100%)	190 (100%)	0.037**
**Cirrhosis**	Yes	163 (11%)	38 (5.1%)	125 (17.1%)	
	No	1,316 (89%)	709 (94.9%)	607 (82.9%)	
	Total	1,479 (100%)	747 (100%)	732 (100%)	<0.0001*
**Hepatitis B**	Yes	453 (50.1%)	365 (71%)	88 (22.5%)	
	No	452 (49.9%)	149 (29%)	303 (77.5%)	
	Total	905 (100%)	514 (100%)	391 (100%)	<0.0001*
**Hepatitis C**	Yes	29 (4.7%)	9 (2.7%)	20 (6.8%)	
	No	593 (95.3%)	319 (97.3%)	273 (93.2%)	
	Total	622 (100%)	328 (100%)	293 (100%)	0.021*
**Hepatitis B+C**	Yes	8 (1.4%)	5 (1.6%)	3 (1.1%)	
	No	583 (98.6%)	301 (98.4%)	282 (98.9%)	
	Total	591 (100%)	306 (100%)	285 (100%)	NS*
**Nodule(s)**	= 1	450 (41.4%)	246 (38.3%)	204 (45.8%)	
	>1	638 (58.6%)	397 (61.7%)	241 (54.2%)	
	Total	1,088 (100%)	643 (100%)	445 (100%)	0.015*
**Metastasis**	Yes	430 (30.1%)	244 (33.3%)	186 (26.7%)	
	No	1,000 (69.9%)	489 (66.7%)	511 (73.3%)	
	Total	1,430 (100%)	733 (100%)	697 (100%)	0.007*
**Operation**	Yes	298 (19.3%)	159 (20.4%)	139 (18.2%)	
	No	1,243 (80.7%)	619 (79.6%)	624 (81.8%)	
	Total	1,541 (100%)	778 (100%)	763 (100%)	NS*
**Recurrence** ‡	Yes	132 (44.7%)	76 (48.7%)	56 (40.3%)	
	No	163 (55.3%)	80 (51.3%)	83 (59.7%)	
	Total	295 (100%)	156 (100%)	139 (100%)	NS*

≤44 and >44 groups are defined as patients below or equal age 44 and those above age 44, respectively. Mean values are presented with ± standard errors of the means (SEM). Percentages are expressed as ratio of the total patient population for the considered parameter. *P*-values were obtained by exact simple (*) or multinomial (**) logistic regression analysis, modelling the specific outcomes with age (≤44 groups vs. >44 group) after geographical and seasonal adjustments. NS: not significant.

† Tumor grades were defined as follow: well differentiated (G1), moderately differentiated (G2), and poorly differentiated (G3).

‡ The data on recurrence are presented for patients who developed a new HCC within the following 12 months after surgical intervention.

### 2.2. Hospital Procedure and Monitored Parameters

Individuals suspected to have malignant liver neoplasm were managed through the Department of Abdominal Surgery of INEN. The vital records and medical history of these patients were documented during their first consultation. Detection and preliminary clinical characterization of the tumor were carried out by both physical examination and non-invasive diagnostic imaging, i.e. computed axial tomography and abdominal ultrasounds. Cancer staging was classified according to the TNM and the Barcelona-Clinic Liver Cancer (BCLC) staging systems [9]. Pathologists assessed the type of cancer cells on hematoxylin–eosin-stained liver biopsy sections and scored the grade of tumor (G1–3) according to the recommendations of the American Joint Committee on Cancer [10]. Hepatoblastoma and intrahepatic cholangiocellular carcinoma cases were not included in the study.

Liver function tests were conducted by serum quantitation of gamma-glutamyl transpeptidase, alkaline phosphatase, total and direct bilirubin, albumin, and alanine and aspartate transaminases. Tumor marker alpha-fetoprotein (AFP) was monitored for its serum concentration (seroAFP) by radioimmunoassay (Roche) during the patient’s hospital stay. In parallel, infections with HBV and hepatitis C virus (HCV) were assessed from serum of HCC patients by electrogenerated chemiluminescence assay using antibodies against HBV surface antigen (HBsAg), anti-HBs antibody, anti-HBc IgG and IgM, HBe antigen, and anti-HCV antibody (all from Roche).

Patients with a Child-Pugh score of A were treated by anatomic liver resection, i.e. systematic removal of the tumoral liver segments confined by portal branches, to ensure tumor-free margins [11]–[13]. The surgical report recorded the size, location, and appearance of the tumor, as well as additional pathological observations (e.g. cirrhotic, multinodular, and/or metastatic status). Patient survival was followed-up prospectively by periodic medical consultations or phone interviews.

### 2.3. Statistical Analysis

Age distribution was analyzed using a Gaussian mixture model for detecting bimodality and calculating the bimodality index (BI) and the moment of mixture (MM) [14]–[16]. Description of variables was reported by median (inter-quartile range) or mean (95% confidence interval) depending on the normality of the distribution assessed by the Shapiro-Wilks test [17]. Bivariable comparisons were conducted by parametric or non-parametric tests depending on the normality of the distribution. A multivariable analysis was performed in order to identify the age-adjusted effect. Linear regressions were used to model the dispersion of normally distributed continuous variables. Simple exact and multinomial logistic regressions were used to model the outcomes with dichotomized age (i.e. ≤44 vs. >44) as the predictor after patients’ geographical precedence and seasonal adjustments. Multidimensional outliers were assessed with the Hadi test and nested models were compared with the likelihood ratio test [18]. The tumor size was transformed with the square root function in order to make it normally distributed. Patient survival rates were analyzed using the Kaplan–Meier method and the log-rank test was used to compare survival distributions [19]–[21]. Statistical analyses were performed with a 5% significance level using Stata Statistical Software: Release 11 (College Station, TX: StataCorp LP) and SPSS 13.0 predictive analytics software (SPSS Inc.). Patient places of origin (birthplace) were spatially aggregated according to a standard deviation classification of mean age, and choropleth mapping was performed using Philcarto mapping software [22].

## Results

**Figure 1:**
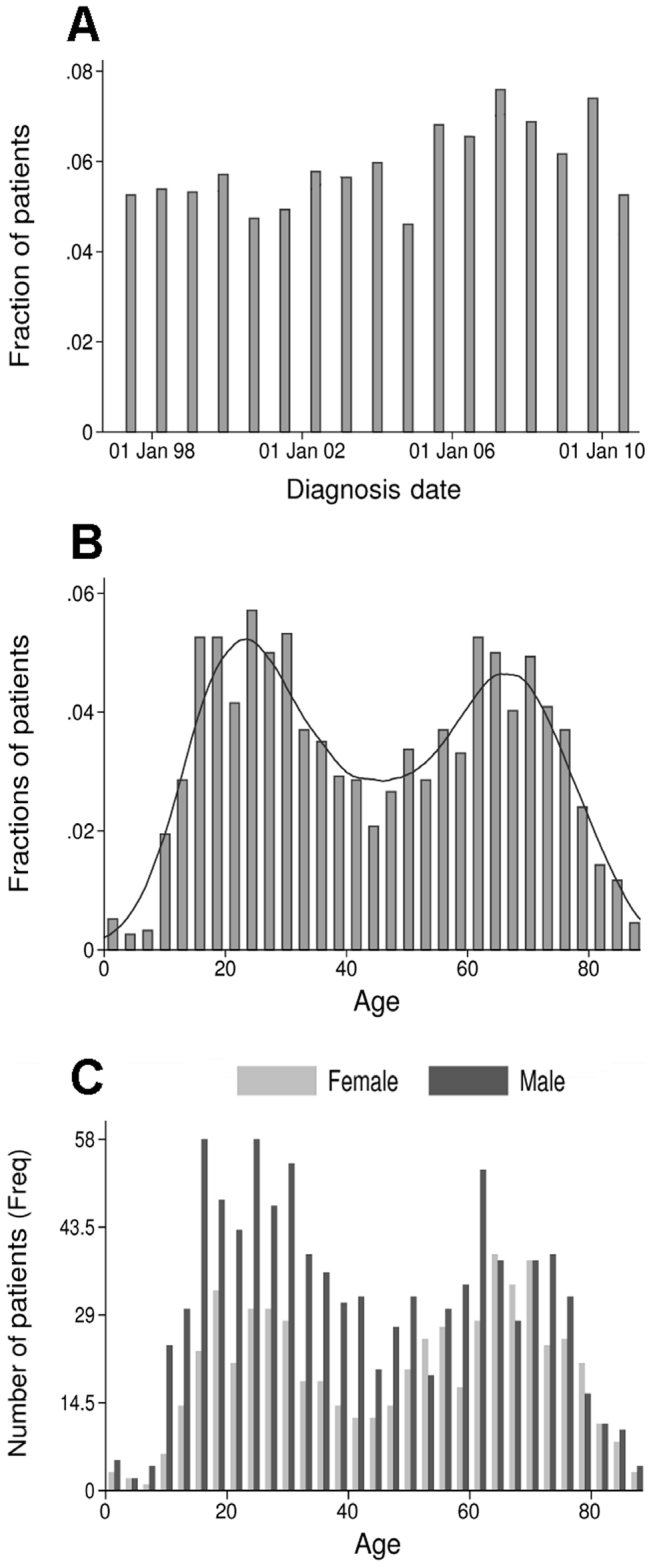
Distribution of the patients admitted at INEN with HCC from January 1997 to December 2010. Histograms show the distribution of patients over the period in function of their diagnosis date (**A**), age at the time of diagnosis (**B,C**), and gender (**C**). X-axes display date (annual interval) (**A**) and age (3-year interval) (**B,C**); Y-axes display fraction (**A,B**) and frequency (Freq) (**C**) of the total number of patients (N = 1,541). Straight line represents the histogram curve fitting with a Gaussian mixture function (BI = 1.95; MM: 44.8) (**B**). Female and male patients are represented in light and dark greys, respectively (**C**).

The INEN admitted an average of 112 HCC cases per year (Figure 1A). The mean age of the patients was 44.8±21.9 years old (Table 1). The age distribution of the HCC patients displayed bimodality with two frequency modes corresponding to two distinct age-based subpopulations (BI = 1.95) (Figure 1B,C and Table 1). The MM of the two modes corresponding to the in-between age was calculated at 44.8 years old (Figure 1B). The patient distribution for the two modes was 778 for the group younger or equal to age 44 (≤44 group), and 763 the group older than age 44 (>44 group) (Figure 1B,C and Table 1). The respective mean and median ages were 25.5±9.5 and 25 in the ≤44 group, and 64.5±10.4 and 65 in the >44 group (Figure 1B and Table 1). The overall sex ratio was 1.6, with 61.1% and 38.9% of men and women, respectively (Table 1). The age-specific sex ratios were significantly different with 2 and 1.2 in the ≤44 group and the >44 group, respectively (*P*<0.0001) (Figure 1C and Table 1).

**Figure 2:**
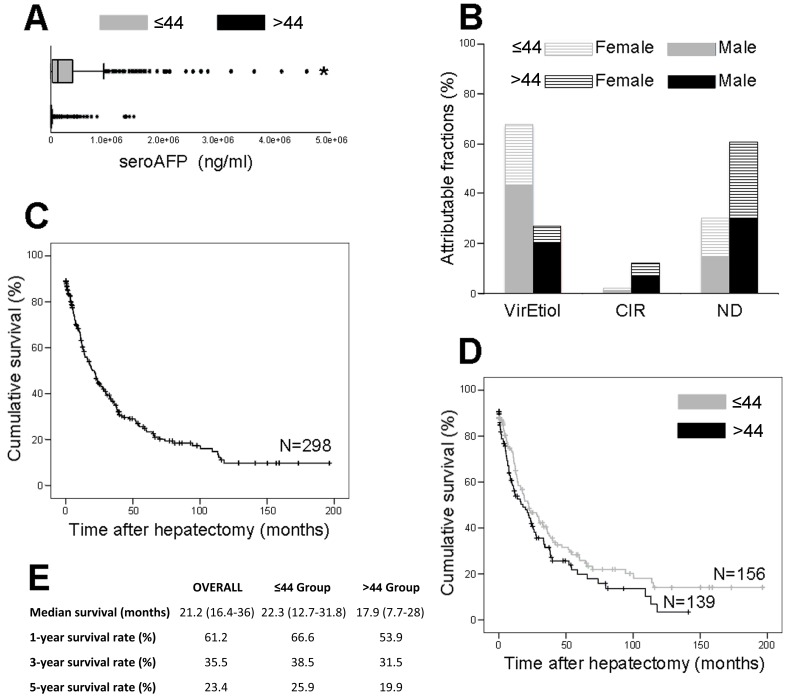
Pathophysiological parameters associated with HCC in the two age-based patient populations. (**A**) Box plots show quartile distribution of seroAFP in ng/ml in ≤44 group (grey box; N = 631) and >44 group (black box; N = 558). *P<0.0001 vs. >44 group (i.e. adjusted multiple linear regression analysis). (**B**) Estimated fractions (%) of HCC attributable to HBV and HCV etiologies (VirEtiol) or non-viral cirrhosis (CIR), or non-attributable to *bona fide* risk factor (ND) in ≤44 females (hatched grey) and males (solid grey) and >44 females (hatched black) and males (solid black). (**C**) Overall Kaplan–Meier survival curve for patients selected for anatomic liver resection (N = 298). (**D**) Age-based Kaplan–Meier survival curves for the ≤44 group (grey curve; N = 156) and the >44 group (black curve; N = 139). (**E**) Kaplan–Meier survival rates (%) monitored for the 2 age-based populations at 1, 3, and 5 years following hepatectomy. Median survival is presented as month (upper – lower interquartile range).

Patients were hospitalized with an average tumor size of 12.3±5.6 cm-diameter. The age-specific average tumor size was 13.1±5.7 and 11.4±5.4 cm-diameter for the ≤44 and >44 groups, respectively. The tumor size was transformed with the square root function in order to make it normally distributed for linear regression analysis. ≤44 and >44 groups displayed significant difference in tumor dimension (*P*<0.0001). A higher prevalence of distant metastases was recorded in the ≤44 group (*P* = 0.007), while multinodular HCCs were preponderant in the >44 group (*P* = 0.015) (Table 1). A significant ratio of undifferentiated or poorly differentiated tumors with high grades (G3) among the ≤44 group compared to the >44 group (*P* = 0.037) (Table 1). The tumor size standardization demonstrated a drastic 6.7 fold elevation of AFP in the ≤44 group with 29,124±3,382 ng/ml/tumor-cm, compared to the 4,331±311 ng/ml/tumor-cm of the >44 group (*P*<0.0001) (Figure 2A). Although it remained remarkably low in both subpopulations, the prevalence of associated cirrhosis was significantly higher in the >44 group, showing 17.1% vs. 5.1% in the ≤44 group (*P*<0.0001) (Figure 2B and Table 1). Linear regression analyses on the quantitation of gamma-glutamyl transpeptidase, alkaline phosphatase, bilirubin, albumin, and transaminases did not demonstrated neither deviant values nor significant difference between the ≤44 and >44 groups (*P*>0.05).

While the rate of HBV infection was substantially higher in the younger population (*P*<0.0001), the HCV infection rate was significantly higher in the older one (*P* = 0.021) (Figure 2B and Table 1). Logistic regression analyses showed that cancer history in both nuclear and extended families was not a discriminating trait between the two age-based subpopulations (*P* = 0.123). Overall, 46.2% of patients did not present any associated risk factor, such as cirrhosis or hepatitis (Figure 2B).

Among the 298 patients selected for surgery, 44.7% developed a recurrence within 12 months following anatomic liver resection (Table 1). The survival rate at 5 years following the intervention was not significantly different between the two subpopulations, with 25.9% and 19.9% for the ≤44 and >44 groups, respectively (*P* = 0.104) (Figure 2C–E and Table 1).

**Figure 3:**
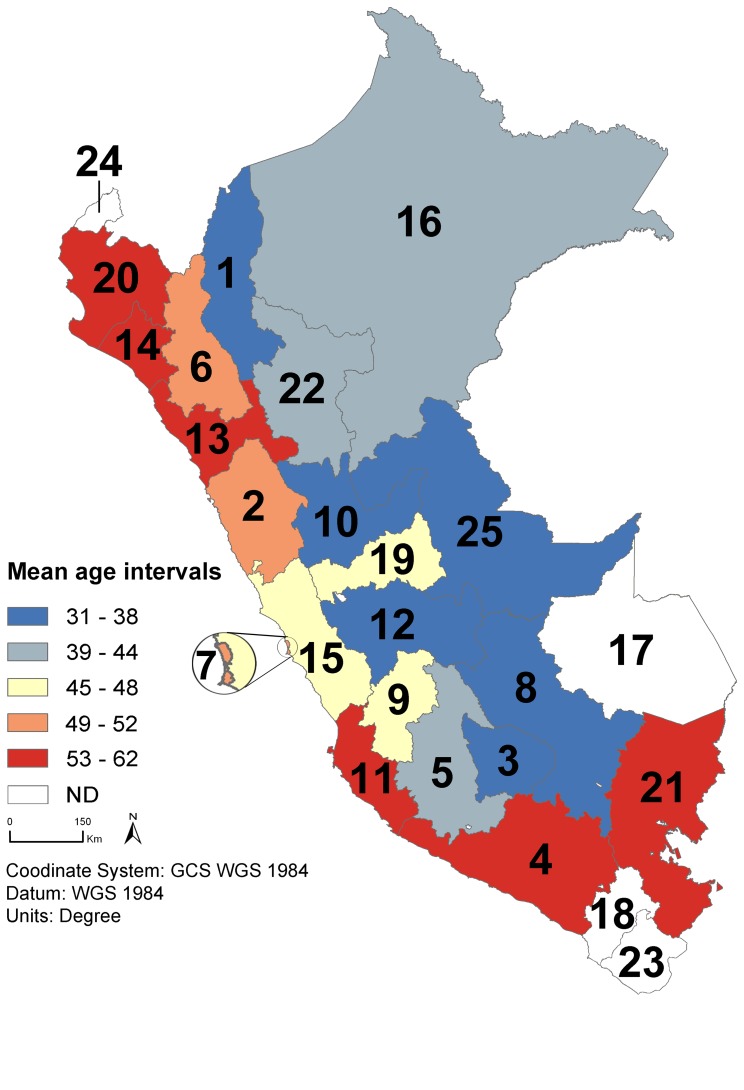
Regional disparities in mean age of Peruvian patients with HCC. The map was generated using the incidence rates of patients admitted at INEN with HCC between 1997 and 2010. The regions are classified as follow: (1) Amazonas (N = 20; mean age: 36.2±20.3); (2) Ancash (N = 117; 49.7±22.3); (3) Apurimac (N = 121; 31.6±17.9); (4) Arequipa (N = 39; 56.6±22.3); (5) Ayacucho (N = 161; 39.5±20.6); (6) Cajamarca (N = 36; 50.2±19.4); (7) Callao (N = 21; 52.3±20.7); (8) Cuzco (N = 96; 33.8±16.6); (9) Huancavelica (N = 32; 45.3±23.9); (10) Huanuco (N = 68; 37.2±20.8); (11) Ica (N = 43; 56.1±19.3); (12) Junin (N = 180; 37.2±19.9); (13) La Libertad (N = 68; 61.6±19.3); (14) Lambayeque (N = 53; 56.8±19.8); (15) Lima (N = 289; 48±21.5); (16) Loreto (N = 21; 42.7±20.8); (17) Madre de Dios (N = 2; ND); (18) Moquegua (N = 5; ND); (19) Pasco (N = 29; 47.3±19.6); (20) Piura (N = 64; 58.2±18.5); (21) Puno (N = 18; 55.4±13.7); (22) San Martin (N = 26; 43.3±19.6); (23) Tacna (N = 4; ND); (24) Tumbes (N = 3; ND); and (25) Ucayali (N = 25; 38.2±18.8). Mean age intervals (years) are established as follow: [31]–[38] (dark blue); [39]–[44] (light blue); [45]–[48] (yellow); [49]–[52] (pink); and [53–62] (red). ND: Mean age was not determined because the cohort of patients admitted at INEN during the period and originating from these regions was too small. Administrative regional limits from the Peruvian National Institute of Statistics and Informatics (INEI).

Mapping patients’ place of origin revealed geographic disparities in age-based HCC development (Figure 3). While the youngest mean ages were recorded in the inland Andean regions with the lowest value in Apurimac region [31.6±17.9 years old (Figure 3, region number 3)], the oldest mean ages were monitored in the coastal and southern regions, culminating in La Libertad region [61.6±19.3 years old (Figure 3, region number 13)].

## Discussion

This study sought to characterize the clinical-epidemiological situation of HCC in Peru, the country in South America with highest incidence of primary liver cancers [1]. The study was conducted by retrospectively analysing the clinical records of patients with HCC hospitalized at INEN between 1997 and 2010. During this period, INEN admitted over 100 cases of HCC per year, about 25% of the national incident cases of primary liver cancer according to the GLOBOCAN 2008 estimates (Figure 1A and Table 1) [1]. Patients hospitalized with HCC were mostly low- and medium-income patients with poor health care coverage. Positioned at a central hub for neoplasm treatment in Peru, INEN received patients from all 25 administrative regions of the country providing an *ad rem* nationwide sampling perspective (Figure 3).

It is generally admitted that HCC occurs mostly in men over 40 years old [1], [3], [5]. In high incidence areas of West Africa and East Asia, age-specific incidence distributions are unimodal and increase after 40 years of age to culminate around 55 [5]. In our study, the age-specific distribution of the patients exhibited an astonishing bimodal pattern corresponding to two distinct age-based subpopulations (Figure 1B,C). This unusual feature prompted us to analyze subsequently the cohort according to age by dividing the series in two distinct subsets below and above age 44, which corresponds to the MM of the bimodal Gaussian distribution. Between 1997 and 2010, the proportions of these two age-based subpopulations were similar with the ≤44 and >44 groups representing respectively 50.5% and 49.5% of the overall patient population (Table 1). A careful examination of the ≤44 and >44 group distributions on an annual basis did not show any noticeable evolution of age repartition over the period; indicating that the presence of young HCC patients is a constitutive feature of the disease in Peru. While the older group was in keeping with the age range observed over the world, the ≤44 group with a mean age of 25 corresponded to an epidemiologically deviant population of patients with regard to the world standard of HCC development [1]–[6]. A study conducted in Mozambique in 1985 showed a significant HCC incidence among young patients, however they were included within a unimodal age distribution and a clear distinction between two age-based subpopulations was not established [23].

In Peru, a large majority of patients were diagnosed with a tumor exceeding 10 cm in diameter. The consistent detection of advanced HCC may reflect a restricted access to health care in the country [8]. However, this situation is most likely due to several features intrinsic to the type of liver disease that affects Peruvian patients. Amazingly, proportions of HCC patients with and without liver cirrhosis in Peru (11% and 89%, respectively) are inverted when compared to the European and North American standards, in which rates of up to 80–90% of cirrhosis-associated HCC are observed (Table 1) [3]–[6]. In addition, almost half the patients lack a *bona fide* risk factor (Figure 2B). Both characteristics imply that in most Peruvian cases, HCC is diagnosed without any prodromes or long-lasting liver disease enabling the prediction of an ongoing tumorigenic process. As Peruvian HCC develops essentially in healthy liver, the clinical symptoms commonly associated with an advanced malignant tumor represent the inaugural signs of hepatic neoplasia in Peru. Such a situation is exceptional in Asia, Europe, and North America, whereas it is the presentation of a minority of HCC cases in Africa [2]–[6], [9].

Current staging systems, e.g. the TNM and BCLC classifications, seem to be inadequate for the Peruvian context of HCC, as they have been validated by surgically oriented groups from the northern hemisphere treating patients in an utterly different pathophysiological context [9], [24], [25]. The generally late detection of HCC in Peru resulted in merely 20% of the ≤44 patients being selected for tumor resection over the period (Table 1). The aggressiveness of HCC in younger patients was illustrated by the higher percentage of early recurrence and lower 5-year survival rate among ≤44 group of patients which underwent resection of large HCC, as compared to previous studies (Figure 2D,E and Table 1) [26]–[30]. Such astounding figures highlight the urgent need for a population-wide screening program of HCC in Peru.

Interestingly, the ≤44 and >44 groups displayed age-specific pathophysiological characteristics (Figure 2A,B and Table 1). The ratios of affected men to affected women were significantly higher in the younger population than in the older one with 2 and 1.2, respectively (Figure 1C and Table 1). Withal, sex ratio in both subpopulations appeared to be more balanced than those observed elsewhere in the world (up to 7 in selected populations) [1], [26]. Besides, metastasis-associated HCC occurred significantly more in the younger population and development of intrahepatic multinudolar tumors took place preferentially in the older group (Table 1). Albeit surprisingly low, the rate of cirrhosis was higher in the >44 group than in the ≤44 group with 17% and 5%, respectively (Figure 2B and Table 1) [3]–[6]. The biological base of such situations remains unexplained and it is difficult to determine whether the outcome is due to an explosive tumor process that does not require chronic insult of liver tissue to develop. Otherwise, it may be the consequence of a particular resistance of the Peruvian populations to cirrhogenic conditions.

The seroAFP was exceedingly high in the ≤44 group compared with the >44 group (Figure 2A). The mean of AFP concentration for the ≤44 group reached 332,000 ng per millilitre, while AFP biosynthesis is usually augmented during HCC up to hundreds or thousands ng per millilitre [31]–[33]. AFP is transiently produced during embryonic development in the establishment of the endodermal cell lineages (e.g. hepatocytes) and its expression is reactivated during HCC development [31]–[37]. Carcinogenesis shares underlying molecular mechanisms with cellular differentiation [38]–[41]. Homeotic genes that function in cell specialization by regulating differentiation marker expression such as AFP are misregulated in cancer [42]–[45]. The tremendously high seroAFP monitored in the ≤44 group therefore supports the hypothesis that aberrant activity of the genes involved in the cell identity and homeostasis drives hepatocarcinogenesis in this patient subpopulation. This hypothesis is reinforced by the predominance of undifferentiated and poorly differentiated hepatic cancer cells encountered in the ≤44 group (Table 1).

Multiple factors can be responsible of HCC development, including genetics, infectious agents, comorbid conditions, and environmental exposures [6], [46]–[51]. Each of these factors has a specific impact on the development of liver cancer and influences the natural course of the neoplasm, both at cellular and pathophysiological levels [33], [51]. As in western Africa and eastern Asia, the major of risk factors identified in Peru is the infection with HBV (Figure 2B) [3]–[6]. A large majority of HBV strains found in Peru belongs to subgenotype F1 and present peculiar mutations and deletions in the viral polymerase and PreS regions, respectively [52]. However, the tumorigenic potential of these strains remains unknown [52]. Classically, the age at which HCC develops is closely related to the age of acquisition of infection with hepatitis and to the rate of viral replication [5]. In a case of an early infection with HBV and an active viral replication (e.g. East Asia), the age-specific distribution of HCC starts to increase at the age of 50 without reaching a plateau [5]. In the case of a later infection with HBV and a rapid decline of viral replication after adolescence (e.g. West Africa), the age-specific distribution of HCC represents a single peak starting between the ages of 45 and plateauing thereafter [5], [23]. In cases where HBV infection occurs in adulthood, the HCC is rarely developed before the age of 50 years and the highest age-specific incidence rates are observed in people over the age of 75 years [5]. None of these age distribution patterns were observed for the Peruvian patients admitted at INEN (Figure 1B). Moreover, the HCC etiology appears to be different between the ≤44 and >44 groups, as the percentage of people over age 44 infected with HBV was significantly lower than in the ≤44 group (Figure 2B and Table 1). In addition, 50% of the patients tested for hepatitis B infection were serologically negative for HBsAg (Figure 2B and Table 1). Thus, while the role of HBV infection as a major etiology of HCC development in Peru is certain, it is not always present and appears, therefore, to be insufficient in and of itself to explain the peculiarities of the explosive tumor process as it is currently observed in Peru.

The particularity of the HCC epidemic is reinforced by the geographical mapping of the patients according to their place of origin. This process resulted in a striking geographical pattern in which the younger patients were concentrated mostly in the inland regions of the Andes, while the coastal regions provided HCC cases with a more elevated age (Figure 3). As a consequence, geographically neighbouring regions could display a 25 years difference with regard to the mean age of HCC patients, as observed for example in both Apurimac/Arequipa and Huanuco/La Libertad tandems (Figure 3; region number 3/4 and 10/13, respectively). At the moment, environmental or ethnogeographical differences that may explain such dramatic variations in tumor presentation remain unknown. In order to confirm this data, the eventuality of a selection effect has to be discarded with a prospective field study. Indeed, the lack of cancer surveillance at the national level and the deficit of information about the patients’ clinical pathway prior to arrival at INEN prevent the accurate evaluation of the real proportions of the ≤44 and >44 HCC populations in Peru. Uncharacterized biasing parameter(s) may result in enhancing the number of ≤44 patients hospitalized at INEN. However, while such selection effect could have impacted quantitatively the perception of the HCC context in our study, the age-specific HCC pathophysiology in the ≤44 and >44 groups clearly marked the distinction between these two subpopulations, suggesting two different age-specific natural histories of HCC in the Peruvian patient population.

The bimodal age-based distribution, the young age of a distinct subgroup, the low rate of associated cirrhosis, the high seroAFP, and the geographical clustering of the cases by age delineate an unusual tumoral process ongoing in the Peruvian population. These findings support the hypothesis that a singular risk factor, environmental, infectious, or genetic, and possibly endemic to the region is driving liver tumorigenesis in a significant proportion of Peruvian HCC patients. Research programs embracing the various aspects of this intriguing disease are required to decipher the biological process ongoing in young Peruvian HCC patients with the aim to generate novel prediction and prevention tools and assays for the local public health authorities.
